# CTHRC1 promotes the progression of clear cell renal cell carcinoma via the PI3K/AKT/GSK3β signaling pathway

**DOI:** 10.1016/j.gendis.2025.101688

**Published:** 2025-05-13

**Authors:** Wei Xie, Zhouyong Fu, Thomas Skutella, Qing Jiang, Ronggui Zhang, Yuanfeng Zhang

**Affiliations:** a Department of Urology, The Second Affiliated Hospital of Chongqing Medical University, Chongqing 400010, China; b Chongqing General Hospital, School of Medicine, Chongqing University, Chongqing 401147, China; c Department of Urology, People's Hospital of Fengjie, Chongqing 404600, China; d Institute for Anatomy and Cell Biology, Heidelberg University, Heidelberg 69120, Germany

Human renal cell carcinoma ranks among the most prevalent forms of cancer.^1^ The predominant subtype, clear cell renal cell carcinoma (ccRCC), constitutes approximately 80% of all kidney cancer cases.^2^ The collagen triple helix repeat containing-1 (CTHRC1) gene encodes an extracellular matrix-associated protein that is up-regulated in various human tumors and is intricately linked to cellular processes such as proliferation, invasion, and metastasis.^3^^,^^4^ It has been demonstrated that CTHRC1 facilitates tumor initiation and progression by modulating the phosphoinositide 3-kinase (PI3K)/protein kinase B (AKT) signaling pathway, thereby enhancing tumor dissemination, invasion, migration, adhesion, and metastasis.^5^ In this study, a multilevel analysis was launched to deeply investigate the role of CTHRC1 in patients with ccRCC by bioinformatics, and as a supplement to it, *in vitro* and *in vivo* experiments were performed to disclose the biological effects of CTHRC1 and explore its therapeutic value. This study demonstrates that CTHRC1 promotes ccRCC progression via the PI3K/AKT/glycogen synthase kinase 3 beta (GSK3β) signaling pathway.

To investigate the expression of CTHRC1 in ccRCC and its association with patient prognosis, data from The Cancer Genome Atlas (TCGA, https://portal.gdc.cancer.gov/) and the GSE53757 dataset were utilized for bioinformatics analysis. Our findings indicated that CTHRC1 expression was significantly up-regulated in ccRCC tissues versus adjacent non-cancerous tissues (Fig. 1A, B). Furthermore, survival analysis revealed that elevated CTHRC1 expression was inversely correlated with the prognosis of patients with ccRCC (Fig. 1C). Additionally, a univariate Cox regression analysis was conducted, revealing that CTHRC1 might serve as an independent risk factor for ccRCC (Fig. 1D).

**Figure 1:**
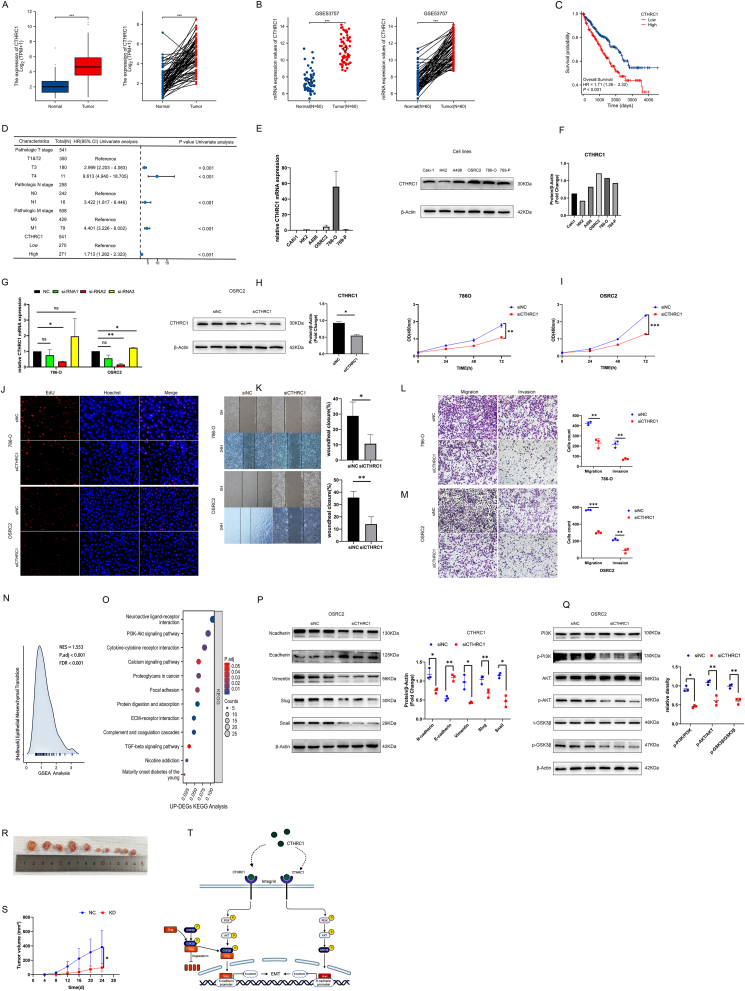
CTHRC1 promotes ccRCC progression by mediating the PI3K/AKT/GSK3β signaling pathway. **(A)** The expression of CTHRC1 among multiple tumors and corresponding normal tissues in the TCGA database. **(B)** Comparison of CTHRC1 expression between tumor tissues and adjacent normal tissues in unpaired and paired samples based on the GSE53757 dataset. **(C)** Correlation between overall survival (OS) and CTHRC1 expression of ccRCC based on the TCGA database. **(D)** Univariate Cox analysis for CTHRC1 in ccRCC. **(E, F)** The mRNA and protein levels of CTHRC1 in ccRCC cell lines, including OSRC2, 786-O, 769-P, A498, Caki-1, and normal kidney cell line HK2 measured by reverse transcription PCR (E) and western blotting (F). **(G)** The knockdown efficiencies of CTHRC1 in 786-O and OSRC2 cells were detected by reverse transcription PCR. **(H)** Western blotting analysis of knockdown efficiency of siRNA2 in OSRC2 cells. **(I, J)** The CCK-8 (I) and EdU cell proliferation assays (J) of CTHRC1-knockdown 786-O and OSRC2 cells. **(K)** Wound healing assays for CTHRC1-knockdown 786-O and OSRC2 cells. **(L, M)** Transwell assay results for cell migration and invasion abilities in CTHRC1-knockdown 786-O (L) and OSRC2 (M) cells. **(N, O)** The hallmark of GSEA analysis (N) and the KEGG enrichment analysis (O) based on the up-regulated differentially expressed genes (DEGs). **(P)** Western blotting analysis of the expression of epithelial–mesenchymal transition (EMT)-related markers, including N-cadherin, E-cadherin, vimentin, slug, and snail, in CTHRC1-knockdown OSRC2 cells. **(Q)** Western blotting analysis of PI3K/AKT/GSK3β main proteins' levels, including PI3K, p-PI3K, AKT, p-AKT, GSK3β, and p-GSK3β, in CTHRC1-knockdown OSRC2 cells. **(R, S)** Effect of CTHRC1 knockdown on tumor volume (R) and growth rate (S) *in vivo*. **(T)** CTHRC1 may serve as a promising molecular target for modulating EMT in ccRCC by regulating the PI3K/AKT/GSK3β signaling pathway. ∗*p* < 0.05, ∗∗*p* < 0.01, and ∗∗∗*p* < 0.001.

To further investigate the biological function of CTHRC1 in ccRCC, its expression was examined in five ccRCC cell lines (Caki-1, A498, OSRC2, 786-O, and 769-P) and one normal kidney cell line (HK2) using reverse transcription quantitative PCR assays (Fig. 1E) and western blotting analysis (Fig. 1F). The results indicated that CTHRC1 expression was elevated in OSRC2 and 786-O cell lines relative to the HK2 cell line. Consequently, OSRC2 and 786-O cell lines were selected for the development of CTHRC1 knockdown models to further explore the biological function of CTHRC1 in ccRCC. Three siRNAs were designed for transient transfection, and the knockdown efficiency was assessed using western blotting and reverse transcription quantitative PCR assays. Among these, siRNA2 demonstrated the highest knockdown efficiency (Fig. 1G, H).

To investigate whether the phenotypes of ccRCC were altered following CTHRC1 knockdown, we conducted a series of experiments using CTHRC1-knockdown OSRC2 and 786-O cell line models. These experiments included CCK-8 (Fig. 1I) and EdU (Fig. 1J) assays to assess cell proliferation, as well as wound healing (Fig. 1K) and transwell assays (Fig. 1L, M) to evaluate cell migration and invasion. Our findings indicated that CTHRC1 knockdown significantly inhibited the proliferation, migration, and invasion of ccRCC cells.

To further elucidate the mechanism underlying CTHRC1 facilitating ccRCC metastasis, we conducted Gene Ontology (GO), Kyoto Encyclopedia of Genes and Genomes (KEGG), and Gene Set Enrichment Analysis (GSEA) enrichment analyses and identified 689 up-regulated and 403 down-regulated differentially expressed genes (DEGs). These DEGs were filtered using criteria of |fold change| ≥ 1.0 and *p* < 0.05, based on RNA sequencing expression profiles obtained from the TCGA database. The hallmark analysis of GSEA was performed using the up-regulated DEGs, and the results indicated a positive association between CTHRC1 and epithelial–mesenchymal transition (EMT) (Fig. 1N). Additionally, KEGG enrichment analysis revealed that the PI3K-AKT pathway was the principal signaling pathway enriched by up-regulated DEGs (Fig. 1O). The results of GO, KEGG, and GSEA analyses for the down-regulated DEGs are presented in Figure S1. To validate these findings, we conducted western blotting assays. The knockdown of CTHRC1 resulted in alterations of EMT-related markers, as evidenced by western blotting assays. Specifically, CTHRC1 knockdown was positively correlated with the mesenchymal markers N-cadherin, vimentin, Snail1 (Snail), and Snail2 (Slug), while exhibiting a negative correlation with the epithelial marker E-cadherin (Fig. 1P). These findings suggest that CTHRC1 plays a role in the EMT by modulating the expression of EMT markers, thereby influencing the migratory and invasive capabilities of ccRCC cells. We then employed western blotting assays to determine whether the levels of total and phosphorylated PI3K and AKT proteins (t-PI3K, t-AKT, p-PI3K, and p-AKT) were altered following CTHRC1 knockdown. The results indicated that CTHRC1 knockdown led to a reduction in the phosphorylation levels of PI3K and AKT proteins (Fig. 1Q). Extensive research has established that the phosphorylation of GSK3β significantly influences the activity of Snai1, and the stability of Snail2 is also associated with GSK3β phosphorylation. Additionally, numerous studies have demonstrated that PI3K and AKT are upstream regulators in the GSK3β signaling pathway. We therefore proposed a hypothesis that phosphorylated GSK3β might be co-regulated with the down-regulation of CTHRC1. Consequently, we investigated whether the levels of GSK3β, both total and phosphorylated (t-GSK3β and p-GSK3β), were reduced upon CTHRC1 knockdown. The findings indicated a significant decrease in GSK3β phosphorylation (Fig. 1Q). These results implied that CTHRC1 may facilitate the PI3K/AKT/GSK3β signaling pathway, thereby promoting the EMT in ccRCC and contributing to the progression of ccRCC (Fig. 1T).

Furthermore, to ascertain the potential role of CTHRC1 in promoting tumor growth in ccRCC, we conducted animal experiments utilizing a nude mouse xenograft model. Ten four-week-old female nude mice were randomly assigned to either the negative control (NC) group or the knockdown (KD) group. Tumor cells were subcutaneously injected into the mice in both the NC and KD groups. Tumor development was initially assessed four days post-injection, with subsequent measurements of tumor size recorded at four-day intervals. In the KD group, CTHRC1 siRNA was administered via local injection into subcutaneous graft tumors. The results demonstrated a significantly reduced tumor volume and growth rate (Fig. 1R, S) in the KD group compared with the NC group. These findings suggest that CTHRC1 could facilitate the tumor growth of ccRCC *in vivo*.

In summary, this study demonstrates that the expression of CTHRC1 is linked to unfavorable prognosis in ccRCC and that CTHRC1 facilitates ccRCC proliferation, migration, and invasion via the PI3K/AKT/GSK3β signaling pathway. These results affirm that CTHRC1 serves as a potential biomarker for evaluating ccRCC progression. Our findings may lay the groundwork for developing diagnostic and therapeutic strategies in ccRCC.

## CRediT authorship contribution statement

**Wei Xie:** Writing – original draft, Visualization, Methodology, Conceptualization. **Zhouyong Fu:** Validation, Software, Methodology, Data curation, Conceptualization. **Thomas Skutella:** Resources. **Qing Jiang:** Resources, Project administration, Data curation. **Ronggui Zhang:** Writing – review & editing, Visualization, Supervision, Investigation. **Yuanfeng Zhang:** Writing – review & editing, Supervision, Resources, Project administration, Investigation, Funding acquisition, Conceptualization.

## Ethics declaration

All animal experiments were reviewed by the Laboratory Animal Management and Use Committee of the Second Affiliated Hospital of Chongqing Medical University (approval no. IACUC-SAHCQMU-2023-0006).

## Funding

This research was funded by the Research Program of the Natural Science Foundation of Chongqing, China (No. cstc2021jcyj-msxmX0484).

## Conflict of interests

The authors declared no conflict of interests.
