# Supplementary material for: CTHRC1 promotes the progression of clear cell renal cell carcinoma via the PI3K/AKT/GSK3β signaling pathway
Source: Genes Dis. 2025 May 13;13(2):101688. doi: 10.1016/j.gendis.2025.101688 (PMC12596567; doi:10.1016/j.gendis.2025.101688)
Supplement: Multimedia component 1 [file mmc1.docx]

**Supplementary Materials**

Supplementary Materials and Methods

The TCGA and GEO database analysis

RNA sequencing (RNA-seq) expression profiles and clinical data for 613 renal clear cell carcinoma (KIRC) tissues along with 72 paracancerous tissues were downloaded from the database of The Cancer Genome Atlas (TCGA, <https://portal.gdc.cancer.gov/>). Besides, the GSE53757 set of data as a supplement to the TCGA-KIRC data were downloaded from the Gene Expression Omnibus (GEO) database (<https://www.ncbi.nlm.nih.gov/geo/>). The R Studio 3.6.3 was used to analyze the expression of CTHRC1 in KIRC, based on HTSeq-fragment per kilobase per million (FPKM) data downloaded from TCGA. RNA-seq data in FPKM format were converted to transcripts per million reads (TPM) with the purpose of analyzing the expression profiles. Used to compare unpaired samples was the Mann-Whitney U test.

With the purpose of performing a survival analysis, a large number of CTHRC1 data were downloaded from the TCGA. Furthermore, survival, univariate, and multivariate Cox analyses were performed based on the downloaded RNA-seq data and clinical data from TCGA database. The cutoff values for high and low CTHRC1 expression were the median expression values. Several clinical features, including the grade of pathological and histological, were studied. The KIRC data were divided into two groups based on the median CTHRC1 expression. Used the "limma" package to distinguish differentially expressed genes (DEGs) ^1^. The thresholds chose for differential mRNA expression were |Fold Change (FC)| > 1.0, and p < 0.05. In order to study oncological features associated with CTHRC1, functional enrichment analyses of DEGs were performed, including Gene Ontology (GO), Kyoto Encyclopedia of Genes and Genomes (KEGG), and gene set enrichment (GSEA) analyses. The R packages clusterProfiler^2^ and ggplot2 were used.

Cell culture in vitro

One normal cell line HK2 and five cancer cell lines including OSRC2, Caki-1 (ProCell, China), 786-O, A498 and 769-P, were cultured with the medium of RPMI-1640 (Gibco, Billings, MT ,US) containing ten percent concentration of fetal bovine serum (FBS) in a 5 percent carbon dioxide cell culture incubator (37℃）.

Transfection in vitro

The siRNA interference sequence was designed by Tsingke Biotechnology Co., Ltd. (Beijing, China). with the following specified sequences: CTHRC1 siRNA#1 (GUGGACCUGUAUAAUGGA), CTHRC1 siRNA#2(GCUGUCAGCGUUGGUAUUU), and CTHRC1 siRNA #3 (UCGCACUUCUUCUGUGGAAA). When seeded OSRC2 and 786-O cells densities reached 70%‒80%, a mixture of siRNA (containing 125 μl Opti-MEM (Gibco, USA), 100 pmol siRNA, and 4 μl Lipo-8000(Beyotime, China) per well) was used to transfect cells. The plates were incubated and the medium of the six-well plates was changed 6 hours later. The efficiency of the CTHRC1 knockdown was validated by western blotting (WB) after 96 hours and real-time quantitative PCR (RT-qPCR) 48 hours later.

qRT-PCR and Western blot

Total RNA from 786-O and OSRC2 cells which were transfected with siRNA was extracted using SteadyPure Rapid RNA Extraction Kit (AgBio, China) and its concentration was determined using a NanoDrop2000 spectrophotometer (Thermo Scientific, Waltham, MA, USA). The PrimeScript™ RT Kit (Kusatsu, JPN) was used to complete RT-PCR experiment（the annealing temperature is 60℃, and the number of cycles was set at 40）. The CFX Connect System (a two-step PCR method, Bio-Rad, Hercules, CA, USA) was used to amplify previously reversed transcription products with the help of SYBR Green™ Premix Ex Taq™ II (Takara). Tsingke Biotechnology Co., Ltd. (Beijing, China) was chosen to synthesize the primers for CTHRC1 and β-actin. The data obtained by RT-qPCR experiments were statistically analyzed by the 2-ΔΔCt method. The sequences of the CTHRC1 and internal reference primers were chosen as follows: CTHRC1(Forward (F), GTGGCTCACTTCGGCTAAAAT; Reverse (R), CACTAATCCAGCACCAATTCCTT) and β-actin: (F, ACAACTTTGGTATCGTGGAAGG; R, GCCATCACGCCACAGTTTC).

The total protein from cell samples were extracted with the extraction solution, which was prepared using RIPA lysis buffer and phenylmethylsulfonyl fluoride (PMSF) and the protease and phosphatase inhibitor in a ratio 100:1:1. After using the BCA Protein Assay Kit (Solarbio, Beijing), the absorbance of the samples was measured, and the concentration of the total protein were calculated using a bovine serum albumin (BSA) standard curve. About 20–40 μ g protein lysates were separated by SDS-PAGE in 10% Bis-Tris acrylamide pre-cast gels (Beyotime), and electro-transferred to immunoblot PVDF membranes (Millipore, USA). After blocking with 1× Protein Free Rapid Blocking Buffer, polyvinylidene difluoride (PVDF) membrane was soaked in an appropriate amount (1:1000) of primary antibody and incubated 24 h at 4 °C. Then the secondary antibody was incubated with the PVDF membranes for 1 h. The purchasing information of antibodies are as follows: Anti-β-Actin (Cat#60008-1-Ig, Proteintech), Anti-CTHRC1 (Cat#16534-1-AP), Anti-N-cadherin (Cat#22018-1-AP, Proteintech), Anti-E-cadherin (Cat#20874-1-AP, Proteintech), Anti-Vimentin (Cat#60330-1-Ig, Proteintech), Anti-Snail (Cat#13099-1-AP, Proteintech), Anti-Slug (Cat#9585T, CST), Anti-AKT1/2/3(Cat#T55561S, Abmart), Anti-PI3K (Cat#4257T, CST), Anti-GSK3β(Cat#ab32391, Abcam), Anti-pAKT1/2/3 (Cat#ab192623, Abcam), Anti-pPI3K (Cat#GTX132597, Gene Tex), Anti-pGSK3β (Cat#310010, ZENBIO). The CLINX ChemiScope S6 (Shanghai) was used to observe the protein bands with enhanced chemiluminescence (ECL) (SS1701, Zhongguan).

Cell proliferation assays

CCK8 and EdU assays were chosen to determine whether CTHRC1 expression could have a positive effect on the proliferation capacity of ccRCC. Briefly, a 96-well plate was seeded with 3000 OSRC2 or 786-O cells per well, and when the cell density reached about 90%, the CCK8 assay was performed with the reagent (APExBIO, Houston, TX, USA). The Varioskan LUX Multimode microplate reader (Thermo, USA) was chosen to measure the absorbance at 450nm. EdU staining was performed with the Cell-Light EdU Apollo Kit (RiboBio, Guangzhou). The fluorescence of OSRC2 and 786-O cells pictures were captured under an inverted fluorescence microscope with EdU (Cy5) and nuclear (DAPI) fluorescent stains.

Wound healing assay

OSRC2 and 786-O cells were seeded onto a six-well plate and were divided into negative control (NC) and knockdown (KD) groups. Once the cell density reached approximately 90%, the monolayer was scraped using a suction tip with a volume of 200 μL. PBS was the reagent for washing away residual medium and floating cells. The basal medium was used to replace the medium and to continue cell culture. At the time points of 0h and 24h, an inverted microscope was used to photograph cell scratch healing. The scratch area was calculated by using ImageJ and GraphPad Prism8 was chosen for statistical analyses.

Migration and invasion assay

For migration experiments, a 24-well plate with 500 μL of complete medium per well was selected followed by 200 μL of basal RPMI1640 medium mixed with 5 × 104 tumor cells were added to each well of a 24-well plate filled with an 8-μm Transwell chamber (Jet Biofil, Guangzhou, China) and cultured for 12 h. The chambers were removed after incubation for 12 h. The 4% paraformaldehyde and 0.5% crystal violet were used for fixation and staining, respectively. After wiping off the cells inside the chambers, the chambers were placed under a microscope, and the migrating and invading cells in three fields of view selected at random were counted. For invasion experiments, matrigel was homogeneously layered within the chambers and cultured for 2 h, and then all the cells were inoculated in the Transwell chamber and cultured for 12 h.

In vivo experiments

All animal experiments were reviewed by the Laboratory Animal Management and Use Committee of the Second Affiliated Hospital of Chongqing Medical University (no. IACUC-SAHCQMU-2023-0006) and performed in accordance with relevant guidelines and regulations. The female NU/NU nude mice were four weeks old and were produced by the Charles River Lab. The ten animals were randomly fallen into NC and KD experiment groups (5/group). Mice that developed tumors within the expected time were used as follow-up subjects. Tumor cells from ccRCC were injected subcutaneously into nude mice in the NC and KD groups. A nude mouse transplant tumor model was established, and tumors in the KD group were locally injected with CTHRC1 siRNA (3nmol/20g, twice a week) for observation. The length (L) and width (W) of medium-sized tumors were measured and recorded during each observation. The volume of tumors was calculated with the formula of 1/2 * L * W^2^.

Data analysis

All results were obtained through multiple independent repeated experiments and presented as the mean ± standard deviation (SD), and the principle of independent and three-replicate experiments were observed. Selected students t-test for difference analysis of experimental data between groups. Software GraphPad Prism 8 and Image J were chosen for data analysis. P<0.05 were selected as a statistically significant criterion for judgment (ns, p ≥ 0.05, *, p < 0.05, **, p < 0.01, ***, p < 0.001).

Supplementary Discussion

The CTHRC1 expression is upregulated in many human tumors, including hepatocellular carcinoma, pancreatic, colorectal, lung, and ovarian cancers, and it plays important roles in tumor growth, migration, invasion, and angiogenesis^3–8^. Recent studies have revealed that CTHRC1 is involved in many signaling pathways to promote a variety of human tumors progress. Whereas the role of CTHRC1 in ccRCC hasn’t been fully explored. In this research, we demonstrated the promising of CTHRC1 as a therapeutic target for ccRCC, which was based on bioinformatics analyses as well as experiments in vitro and in vivo.

In this research, analyses of samples from TCGA and GEO showed that the expression of CTHRC1 in normal tissues was much lower than that in ccRCC tissues. Kaplan–Meier survival curves disclosed that the expression of CTHRC1 was negatively related to patients overall survival in ccRCC. To further investigate the character of CTHRC1 in ccRCC, we selected OSRC2 and 786-O cell lines for in vitro experiments and found that the proliferation, migration, and invasion abilities of ccRCC can be inhibited by the knockdown of CTHRC1 to a certain extent. We divided nude mice of the same age, sex, and weight into NC and KD groups and performed subcutaneous tumorigenic experiments. The effect of CTHRC1 knockdown on ccRCC proliferation in vivo was evaluated based on recorded data from nude mouse transplanted tumors in NC and KD groups. We found that knocking down CTHRC1 can inhibit ccRCC proliferation. A functional enrichment analysis was performed to further investigate the cancer-promoting mechanism of CTHRC1 in ccRCC. A KEGG pathway enrichment analysis showed that CHTRC1 probably participated in the pathway of PI3K/AKT and mainly involved in cell adhesion. GSEA analysis showed that CTHRC1 was closely related to the EMT. Then, the Western blotting experiment was chosen to verify the results of the GSEA analysis, and WB results showed that CTHRC1 had a positive correlation with the mesenchymal markers (e.g., N-cadherin,Snail,Slug, and Vimentin) but was negatively related to the epithelial marker (E-cadherin). These results preliminarily demonstrated a link between CTHRC1 and the EMT. In a previous study, Ni et al. demonstrated that CTHRC1 overexpression promotes EMT in colorectal cancer^9^. Liu et al. demonstrated that the knockdown of CTHRC1 inhibits EMT and cell migration^10^. Zhang et al. revealed that CTHRC1 was correlated with the liver metastasis of colorectal cancer. Therefore, we speculated that CTHRC1 may be involved in the EMT to promote ccRCC migration and invasion.

EMT, a particularly charismatic cellular process, has been reported to be positively associated with metastasis and drug resistance in a variety of tumors ^11^. In addition, EMT progression is associated with multiple pathways, for instance, PI3K-AKT, JNK, and JAK-STAT^12^. These signaling pathways create conditions for the metastasis of cancers by regulating the EMT independently or co-regulating it. In this study, a KEGG enrichment analysis showed that CTHRC1 is probably related to the pathway of PI3K-AKT, indicating that CTHRC1 may participate in this pathway to regulate EMT and promote the metastasis of ccRCC. Multiple transcription factors, including ZEB1, snail, and Slug were involved in EMT process, which have been reported by multiple studies^13–15^. In particular, they mainly participate in the activation of early EMT ^16^. Multitude researches suggested that Snail has an indispensable role in regulating the EMT and the metastasis of ccRCC^17–20^. Snail and Slug are now known as Snail1 and Snail2, respectively, and snail-induced EMT is mainly achieved by inhibiting E-cadherin^21^. The loss of E-cadherin expression is considered a key event leading to the EMT^22^. In addition to its involvement in neural crest cell migration and mesoderm formation ^23–25^, its elevated expression is associated with cancer recurrence ^26^.Because the transcriptional activity of SNAIL1 is mainly mediated by p-GSK3β, multiple signaling pathways can indirectly affect the transcriptional activity of SNAIL by modulating p-GSK3β ^27^. The GSK3β phosphorylation level is related to Slug expression and is involved in maintaining Slug stability ^28^.The phosphorylated GSK3β can be regulated by PI3K/AKT to affect Snail1’s transcriptional activity ^12^. Therefore, the Western blotting experiment was conducted to verify whether protein expression of these molecules decreased along with the knockdown of CTHRC1. WB results suggested that CTHRC1 may participate in the pathway of PI3K/AKT/GSK3β to regulate the transcription factors Slug and Snail, thereby inducing the EMT. We then plotted the pattern diagram by which CTHRC1 mediating the pathway of PI3K/AKT/GSK3β to promote EMT; however, more follow-up experiments are needed to verify the specific details.

**References**

1. Ritchie ME, Phipson B, Wu D, et al. limma powers differential expression analyses for RNA-sequencing and microarray studies. *Nucleic Acids Res*. 2015;43(7):e47.

2. Yu G, Wang LG, Han Y, He QY. clusterProfiler: an R package for comparing biological themes among gene clusters. *Omics J Integr Biol*. 2012;16(5):284-287.

3. Guo B, Yan H, Li L, Yin K, Ji F, Zhang S. Collagen triple helix repeat containing 1 (CTHRC1) activates Integrin β3/FAK signaling and promotes metastasis in ovarian cancer. *J Ovarian Res*. 2017;10(1):69.

4. Ding X, Huang R, Zhong Y, et al. CTHRC1 promotes gastric cancer metastasis via HIF-1α/CXCR4 signaling pathway. *Biomed Pharmacother Biomedecine Pharmacother*. 2020;123:109742.

5. He W, Zhang H, Wang Y, et al. CTHRC1 induces non-small cell lung cancer (NSCLC) invasion through upregulating MMP-7/MMP-9. *BMC Cancer*. 2018;18(1):400.

6. Harikrishnan K, Prabhu SS, Balasubramanian N. A pan-cancer analysis of matrisome proteins reveals CTHRC1 and a related network as major ECM regulators across cancers. *PloS One*. 2022;17(10):e0270063.

7. Lee J, Song J, Kwon ES, et al. CTHRC1 promotes angiogenesis by recruiting Tie2-expressing monocytes to pancreatic tumors. *Exp Mol Med*. 2016;48(9):e261.

8. Li LY, Yin KM, Bai YH, Zhang ZG, Di W, Zhang S. CTHRC1 promotes M2-like macrophage recruitment and myometrial invasion in endometrial carcinoma by integrin-Akt signaling pathway. *Clin Exp Metastasis*. 2019;36(4):351-363.

9. Ni S, Ren F, Xu M, et al. CTHRC1 overexpression predicts poor survival and enhances epithelial-mesenchymal transition in colorectal cancer. *Cancer Med*. 2018;7(11):5643-5654.

10. Liu J, Li W, Liu S, et al. Knockdown of Collagen Triple Helix Repeat Containing 1 (CTHRC1) Inhibits Epithelial-Mesenchymal Transition and Cellular Migration in Glioblastoma Cells. *Oncol Res*. 2017;25(2):225-232.

11. Pastushenko I, Blanpain C. EMT Transition States during Tumor Progression and Metastasis. *Trends Cell Biol*. 2019;29(3):212-226.

12. Zhang Z, Zhang Y, Zhang R. P4HA3 promotes clear cell renal cell carcinoma progression via the PI3K/AKT/GSK3β pathway. *Med Oncol Northwood Lond Engl*. 2023;40(2):70.

13. Dufourt J, Bellec M, Trullo A, et al. Imaging translation dynamics in live embryos reveals spatial heterogeneities. *Science*. 2021;372(6544):840-844.

14. Huang W, Zhang J, Huo M, et al. CUL4B Promotes Breast Carcinogenesis by Coordinating with Transcriptional Repressor Complexes in Response to Hypoxia Signaling Pathway. *Adv Sci Weinh Baden-Wurtt Ger*. 2021;8(10):2001515.

15. Kurppa KJ, Liu Y, To C, et al. Treatment-Induced Tumor Dormancy through YAP-Mediated Transcriptional Reprogramming of the Apoptotic Pathway. *Cancer Cell*. 2020;37(1):104-122.e12.

16. Peinado H, Olmeda D, Cano A. Snail, Zeb and bHLH factors in tumour progression: an alliance against the epithelial phenotype? *Nat Rev Cancer*. 2007;7(6):415-428.

17. Cai J. Roles of transcriptional factor Snail and adhesion factor E-cadherin in clear cell renal cell carcinoma. *Exp Ther Med*. 2013;6(6):1489-1493.

18. Li Q, Hou L, Ding G, et al. KDM6B induces epithelial-mesenchymal transition and enhances clear cell renal cell carcinoma metastasis through the activation of SLUG. *Int J Clin Exp Pathol*. 2015;8(6):6334-6344.

19. Lin YW, Wen YC, Hsiao CH, et al. Proteoglycan SPOCK1 as a Poor Prognostic Marker Promotes Malignant Progression of Clear Cell Renal Cell Carcinoma via Triggering the Snail/Slug-MMP-2 Axis-Mediated Epithelial-to-Mesenchymal Transition. *Cells*. 2023;12(3):352.

20. O’Mahony FC, Faratian D, Varley J, et al. The use of automated quantitative analysis to evaluate epithelial-to-mesenchymal transition associated proteins in clear cell renal cell carcinoma. *PloS One*. 2012;7(2):e31557.

21. Barrallo-Gimeno A, Nieto MA. The Snail genes as inducers of cell movement and survival: implications in development and cancer. *Dev Camb Engl*. 2005;132(14):3151-3161.

22. Zhao GX, Xu YY, Weng SQ, et al. CAPS1 promotes colorectal cancer metastasis via Snail mediated epithelial mesenchymal transformation. *Oncogene*. 2019;38(23):4574-4589.

23. Guo C, Li S, Liang A, Cui M, Lou Y, Wang H. PPA1 Promotes Breast Cancer Proliferation and Metastasis Through PI3K/AKT/GSK3β Signaling Pathway. *Front Cell Dev Biol*. 2021;9:730558.

24. Ke B, Zeng Y, Zhao Z, et al. Uric acid: a potent molecular contributor to pluripotent stem cell cardiac differentiation via mesoderm specification. *Cell Death Differ*. 2019;26(5):826-842.

25. Reece-Hoyes JS, Deplancke B, Barrasa MI, et al. The C. elegans Snail homolog CES-1 can activate gene expression in vivo and share targets with bHLH transcription factors. *Nucleic Acids Res*. 2009;37(11):3689-3698.

26. Schinke H, Heider T, Herkommer T, et al. Digital scoring of EpCAM and slug expression as prognostic markers in head and neck squamous cell carcinomas. *Mol Oncol*. 2021;15(4):1040-1053.

27. Zhou BP, Deng J, Xia W, et al. Dual regulation of Snail by GSK-3beta-mediated phosphorylation in control of epithelial-mesenchymal transition. *Nat Cell Biol*. 2004;6(10):931-940.

28. Sh K, Wl W, Cy C, et al. GSK3β controls epithelial-mesenchymal transition and tumor metastasis by CHIP-mediated degradation of Slug. *Oncogene*. 2014;33(24).

29. Pyagay P, Heroult M, Wang Q, et al. Collagen triple helix repeat containing 1, a novel secreted protein in injured and diseased arteries, inhibits collagen expression and promotes cell migration. *Circ Res*. 2005;96(2):261-268.

30. Zeisberg M, Neilson EG. Biomarkers for epithelial-mesenchymal transitions. *J Clin Invest*. 2009;119(6):1429-1437.

**Supplementary Figure**

**Figure S1** KEGG and GO analysis of DEGs. The KEGG enrichment analysis of the down-regulated DEGs (A). (B and C) GO enrichment analysis of the up-regulated DEGs (B) and down-regulated DEGs (C).
